# Effects of Interactions between ZnO Nanoparticles and Saccharides on Biological Responses

**DOI:** 10.3390/ijms19020486

**Published:** 2018-02-06

**Authors:** Mi-Ran Go, Jin Yu, Song-Hwa Bae, Hyeon-Jin Kim, Soo-Jin Choi

**Affiliations:** Major of Food Science & Technology, Department of Applied Food System, Seoul Women’s University, Seoul 01797, Korea; miran8190@naver.com (M.-R.G.); ky5031@swu.ac.kr (J.Y.); songhwa29@naver.com (S.-H.B.); kimhj043@naver.com (H.-J.K.)

**Keywords:** zinc oxide nanoparticles, interaction, saccharides, cytotoxicity, uptake, intestinal transport, oral absorption

## Abstract

Zinc oxide (ZnO) nanoparticles (NPs) are widely used as a Zn supplement, because Zn plays a role in many cellular and immune functions but public concern about their potentially undesirable effects on the human body is growing. When NPs are added in food matrices, interactions between NPs and food components occur, which can affect biological systems. In this study, interactions between ZnO NPs and saccharides were investigated by measuring changes in hydrodynamic radius, zeta potential and solubility and by quantifying amounts of adsorbed saccharides on NPs; acacia honey, sugar mixtures (containing equivalent amounts of fructose, glucose, sucrose and maltose) and monosaccharide solutions were used as model compounds. Biological responses of NPs dispersed in different saccharides were also evaluated in human intestinal cells and rats in terms of cytotoxicity, cellular uptake, intestinal transport and oral absorption. The results demonstrate that the hydrodynamic radii and zeta potentials of NPs were highly affected by saccharides. In addition, trace nutrients influenced NP/saccharide interactions and interactive effects between saccharides on the interactions were found. NPs in all saccharides increased inhibition of cell proliferation and enhanced cellular uptake. Oral absorption of NPs was highly enhanced by 5% glucose, which is in-line with intestinal transport result. These findings show that ZnO NPs interact with saccharides and these interactions affects biological responses.

## 1. Introduction

Zinc oxide (ZnO) nanoparticles (NPs) are widely used in a variety of commercial products, such as in ceramics, paints, pigments, cosmetics and food additives [1,2,3]. In particular, Zn plays an important role in many cellular and immune functions, including cell division, cell growth and wound healing and thus, ZnO NPs are commonly applied as Zn supplements or agricultural fertilizers [4,5,6,7,8]. Along with rapid development of nanotechnology and growing concern about the potential toxicity of NPs, many studies have evaluated their toxicity in cell lines and in animals [9,10,11,12,13]. However, in vitro and in vivo results have been contradictory and appear to depend on the materials and experimental conditions used, which raises questions as to whether NPs cause unexpected biological responses or toxicity, related to their large surface area to volume ratios.

Another important aspect to be considered regarding the potential toxicity of NPs concerns possible interactions between NPs and biological matrices. Especially, NP interactions with plasma proteins, such as albumin and fibrinogen, have been extensively studied and the results suggest that adsorption of plasma proteins on the surface of NPs may induce structural or conformational changes of proteins [14,15,16]. On the other hand, when NPs are added to a complex system like food, interactions between NPs and food matrices could occur, which might affect biological responses or induce unexpected toxicity. Recent report emphasized the necessity to assess the synergistic effects of NPs in a complex system when NPs are applied to food [17]. However, this type of research is extremely limited. Possible NP interactions with food components, such as proteins, carbohydrates, lipids, minerals and trace elements, can also influence the physicochemical property of NPs or nutrients and have undesirable effects on the human body. Indeed, ZnO NPs in the presence of vitamin C was found to increase cytotoxicity and to induce liver and kidney injury [18]. Furthermore, it was recently reported that food-grade silica NPs interacted with saccharides, proteins, lipids and minerals, although the interactions were not strong [19]. It was also demonstrated that the oral absorption of food-grade silica and titanium dioxide NPs significantly increased following oral administration to rats, when they were dispersed in albumin or glucose solution [20,21]. More extensive study is, therefore, required to identify and characterize NP interactions with food components and their impacts on biological responses.

The aim of this study was to investigate quantitatively interactions between ZnO NPs and saccharides, the most common components in food. Acacia honey (10%), composed of ~4.24% fructose, ~2.96% glucose, ~0.01% sucrose, ~0.01% maltose and other trace nutrients, was used as a representative saccharide matrix. Sugar mixtures containing equivalent amounts (1%, 2% and 5%) of fructose, glucose, sucrose and maltose were used for comparative study to determine the role of trace nutrients in NP interactions with saccharides. Two monosaccharide solutions (5% fructose and 5% glucose, the main two saccharides in acacia honey) were also examined to investigate interactive effects between different saccharides on the interactions. The interaction effects on biological responses were evaluated in human intestinal cells and rats in terms of cytotoxicity, cellular uptake, intestinal transport and biokinetics.

## 2. Results

### 2.1. Characterization of ZnO NPs in Saccharides

**Figure 1:**
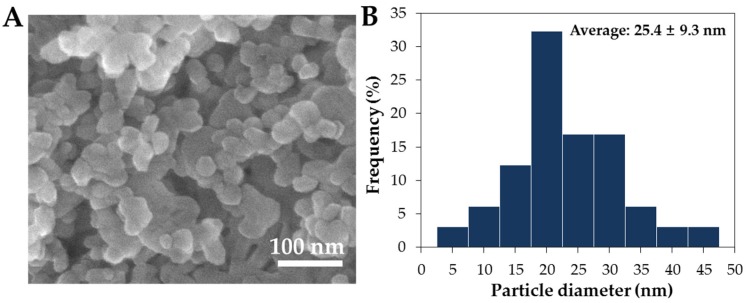
(**A**) Scanning electron microscopic (SEM) images and (**B**) size distribution of ZnO NPs. Particle size distribution was determined by randomly selecting 200 particles from the SEM images.

**Figure 2:**
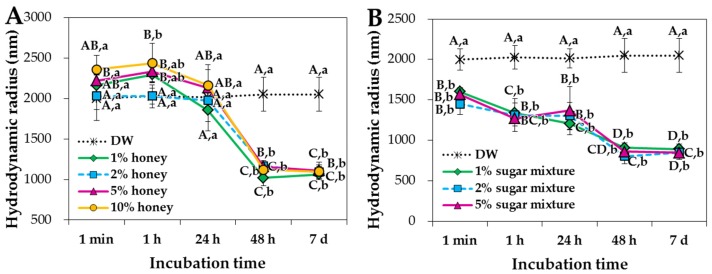
Hydrodynamic radii of ZnO NPs in different concentrations of (**A**) honey and (**B**) sugar mixtures. Different capital letters (A–D) indicate significant differences between incubation times at the same concentration (*p* < 0.05). Different lower-case letters (a,b) indicate significant differences between concentrations after the same incubation time (*p* < 0.05). Sugar mixtures consist of equivalent amounts of fructose, glucose, sucrose and maltose.

**Figure 3:**
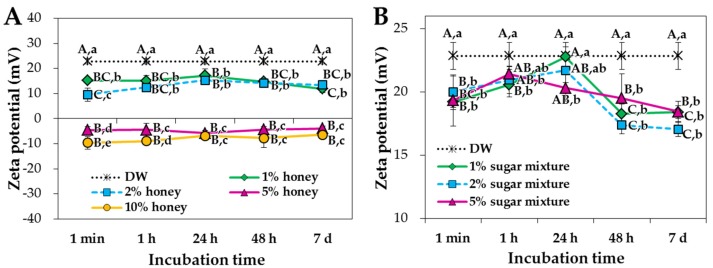
Zeta potentials of ZnO NPs in different concentrations of (**A**) honey and (**B**) sugar mixtures. Different capital letters (A–C) indicate significant differences between incubation times at the same concentration (*p* < 0.05). Different lower-case letters (a–e) indicate significant differences between concentrations after the same incubation time (*p* < 0.05). Sugar mixtures consist of equivalent amounts of fructose, glucose, sucrose and maltose.

The particle shape and mean primary particle size were determined by scanning electron microscopy (SEM), showing spherical shape with ~25.4 ± 9.3 nm (Figure 1A,B). However, the mean hydrodynamic radius of NPs in distilled water (DW), measured by dynamic light scattering (DLS), was 1999.0 ± 32.8 nm (Figure 2 and Figure S1A), indicating their agglomeration or aggregation in aqueous solution. X-ray diffraction (XRD) pattern of ZnO NPs revealed a typical wurtzite structure (Figure S1B). Mean zeta potential of NPs in DW was determined to be 22.9 ± 1.1 mV (Figure 3).

**Figure 4:**
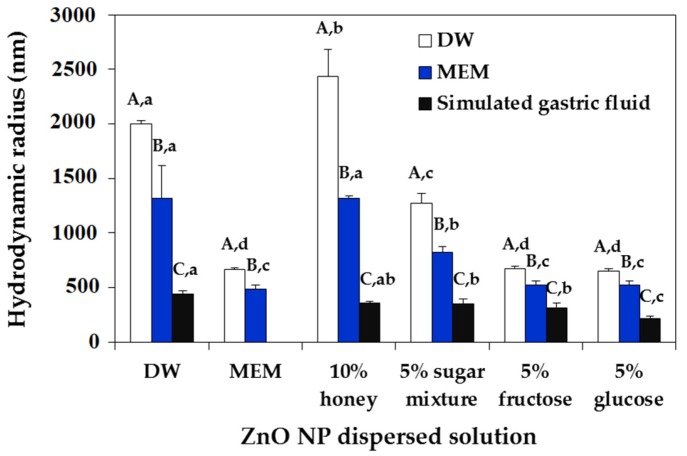
Hydrodynamic radii of ZnO NPs dispersed in DW, minimum essential medium (MEM), or different saccharide solutions for 1 h and then, measured in biological fluids (MEM and simulated gastric fluid) or DW as a control. Different capital letters (A–C) indicate significant differences between DLS measured biological fluids (DW, MEM and simulated gastric fluid) (*p* < 0.05). Different lower-case letters (a–d) indicate significant differences between NP dispersed solutions (DW, MEM, 10% honey, 5% sugar mixture, 5% fructose and 5% glucose) (*p* < 0.05). 5% sugar mixture consists of 5% fructose, 5% glucose, 5% sucrose and 5% maltose.

The hydrodynamic radii of ZnO NPs decreased significantly and gradually as incubation time increased when they were dispersed in honey or sugar mixture solutions at all concentrations tested (Figure 2, Figures S2 and S3). In particular, the hydrodynamic radii dramatically decreased after incubation for 48 h and 7 days in both solutions. The overall hydrodynamic size was not found to be dependent on the concentration of honey or sugar mixture solutions. The highest concentrations of honey and sugar mixture solutions were set at 10% and 5%, respectively, based on consideration of viscosity and solubility. Meanwhile, NPs dispersed in 5% fructose or 5% glucose solutions for 1 h showed dramatic decrease in hydrodynamic radii, as compared with those in DW, 10% honey and 5% sugar mixture, showing 673.5 ± 26.3 and 650.2 ± 25.9 nm, respectively (Figure 4 and Figure S4). These sizes were maintained for 7 days (Figure S5) without statistical difference between fructose and glucose (*p* > 0.05). It is worth noting that the hydrodynamic radii of NPs in DW did not significantly change for 7 days (Figure 2).

**Figure 5:**
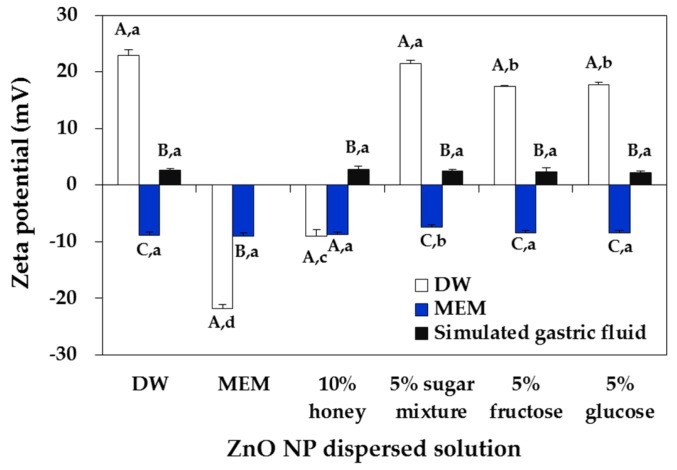
Zeta potentials of ZnO NPs dispersed in DW, minimum essential medium (MEM), or different saccharide solutions for 1 h and then, measured in biological fluids (MEM and simulated gastric fluid) or DW as a control. Different capital letters (A–C) indicate significant differences between DLS measured biological fluids (DW, MEM and simulated gastric fluid) (*p* < 0.05). Different lower-case letters (a–c) indicate significant differences between NP dispersed solutions (DW, MEM, 10% honey, 5% sugar mixture, 5% fructose and 5% glucose) (*p* < 0.05). 5% sugar mixture consists of 5% fructose, 5% glucose, 5% sucrose and 5% maltose.

The zeta potential values of NPs dispersed in honey concentrations of 1% and 2% immediately reduced (became less positive) and became negative at concentration of 5% and 10%, contrary to positive charge (22.9 ± 1.1 mV) in DW (Figure 3A). On the other hand, the zeta potentials of NPs in sugar mixtures slightly changed to less positive direction (Figure 3B). NP dispersed in 5% fructose or 5% glucose for 1 h had the zeta potentials of 17.4 ± 0.4 and 17.7 ± 0.2 mV, respectively, which were significantly lower than those measured in DW or 5% sugar mixture, after incubation for 1 h (Figure 5).

The solubility of NPs was not affected by honey or sugar mixture solutions versus DW, showing 0.7 ± 0.1%, 0.2 ± 0.1% and 0.2 ± 0.1% solubilities in 10% honey, 5% sugar mixture and DW, respectively.

### 2.2. ZnO NP-Saccharide-Biomatrix Interactions

To investigate NP interactions with saccharides in food matrices and their interactions with biofluids, which reflect NP-food matrix-biomatrix corona under experimental cell culture or in vivo oral condition, the hydrodynamic radii and zeta potentials of NPs were measured after particle dispersion in different saccharide solutions for 1 h and then in minimum essential medium (MEM) or simulated gastric fluid (Figure 4 and Figure 5). Figure 4 shows that the hydrodynamic radii of NPs remarkably decreased after further incubation in MEM or gastric fluid in all cases (Figure S4). In particular, the hydrodynamic radii of NPs suspended in 5% fructose or 5% glucose dramatically decreased when they were further incubated in MEM and gastric fluid. More significant decrease in the hydrodynamic radii of ZnO NPs in all saccharides was found after further incubation in gastric fluid as compared with those observed in MEM. On the other hand, no effects of NP-saccharide-biomatrix corona on the zeta potentials of NPs were found (Figure 5, *p* > 0.05).

### 2.3. Quantitative Analysis of Interactions between ZnO NP and Saccharides

**Figure 6:**
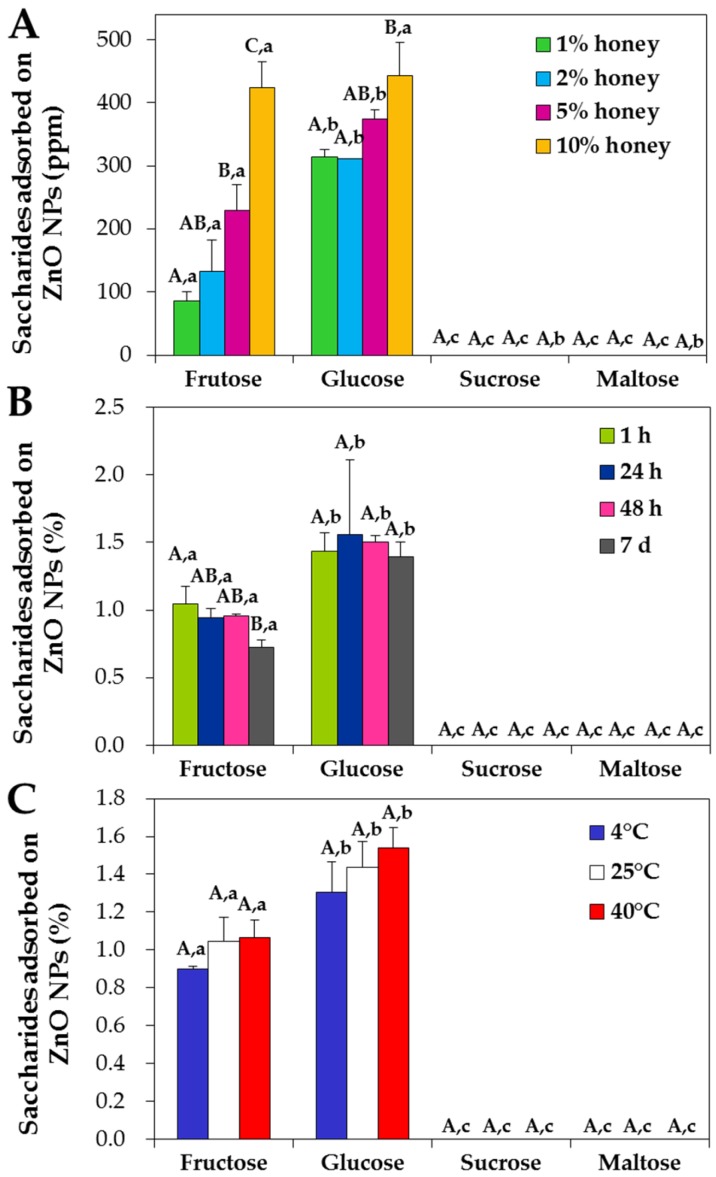
High performance liquid chromatography (HPLC) analysis of the interactions between ZnO NPs and saccharides in acacia honey with respect to (**A**) honey concentrations after incubation for 1 h at 25 °C, (**B**) incubation time in 10% honey at 25 °C and (**C**) temperature after 1 h in 10% honey. Different capital letters (A–C) indicate significant differences in adsorbed amounts of each saccharide (**A**) between honey concentrations, (**B**) between incubation times and (**C**) between temperatures, respectively (*p* < 0.05). Different lower-case letters (a,b) indicate significant differences between saccharide types (**A**) at the same concentration, (**B**) after the same incubation time and (**C**) at the same temperature, respectively (*p* < 0.05).

The interactions between NPs and saccharides in acacia honey were quantified by high performance liquid chromatography (HPLC) with respect to concentration, incubation time and temperature. Figure 6A demonstrates that the interactions between NPs and fructose or glucose in honey increased as concentration increased. In particular, NPs were found to interact most with glucose in honey. However, no adsorbed disaccharides, such as sucrose and maltose, were detected on NPs. Furthermore, neither incubation time (Figure 6B) nor temperature (Figure 6C) remarkably affected NP interactions with saccharides in honey; ~1% and ~1.5% of adsorbed fructose and glucose (of total fructose and glucose in honey), respectively, were found on the surface of NPs.

**Figure 7:**
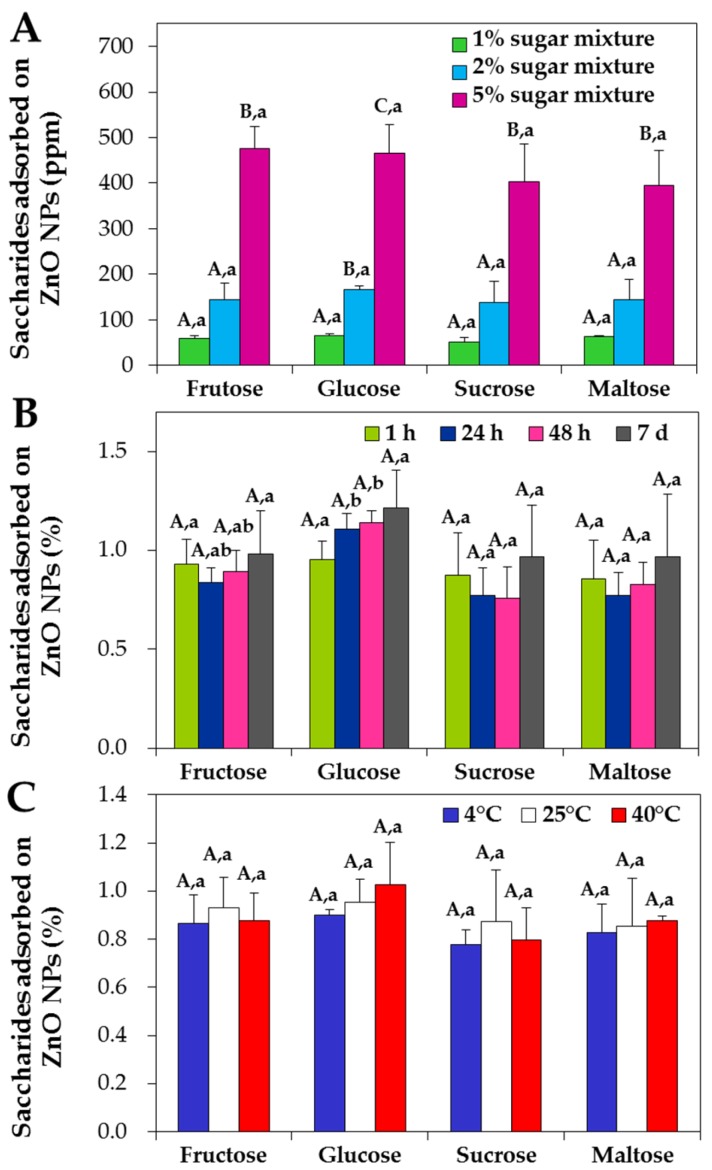
High performance liquid chromatography (HPLC) analysis of the interactions between ZnO NPs and saccharides in sugar mixtures with respect to (**A**) sugar concentration after incubation for 1 h at 25 °C, (**B**) incubation time in 5% sugar mixture at 25 °C and (**C**) temperature after 1 h in 5% sugar mixture. Sugar mixtures contain equivalent amounts of fructose, glucose, sucrose and maltose. Different capital letters (A–C) indicate significant differences in adsorbed amounts of each saccharide (**A**) between sugar mixture concentrations, (**B**) between incubation times and (**C**) between temperatures, respectively (*p* < 0.05). Different lower-case letters (a,b) indicate significant differences between saccharide types (**A**) at the same concentration, (**B**) after the same incubation time and (**C**) at the same temperature, respectively (*p* < 0.05).

When NP interactions with sugar mixtures were quantified, the interactions increased as saccharide concentration increased but no significant differences were found between saccharide types (Figure 7A, *p* > 0.05). Moreover, the interactions were not affected by incubation time (Figure 7B) or temperature (Figure 7C), showing ~1% of interacted amount for all saccharides without statistical differences (*p* > 0.05). On the other hand, when NPs were incubated with 5% fructose and 5% glucose for 1 h, respectively, ~1.3% and ~0.8% were determined to interact with NPs. However, no adsorbed saccharides were detected after washing with DW in any case, indicating that the interactions between NPs and saccharides were weak.

### 2.4. Interaction Effects on ZnO NP Cytotoxicity

**Figure 8:**
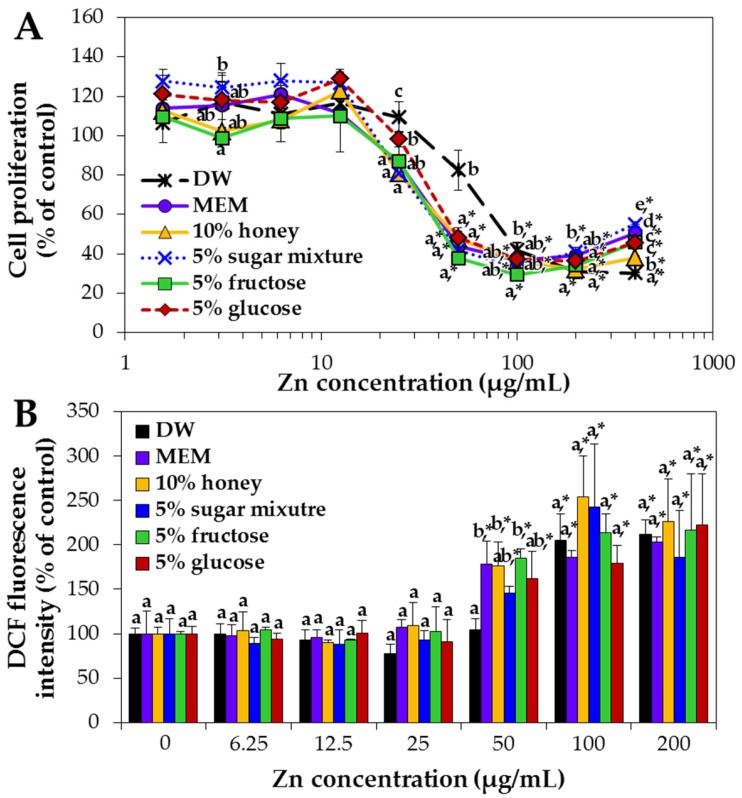
Effects of ZnO NPs suspended in DW, MEM, or different saccharide solutions on (**A**) Caco-2 cell proliferation and (**B**) intracellular ROS generation after 24 h. Different lower-case letters (a–e) indicate significant differences among DW, MEM and different saccharide solutions (*p* < 0.05). * denotes significant difference from NPs in DW as a control (*p* < 0.05).

The effect of the interactions between ZnO NPs and saccharides on cell proliferation was evaluated in human intestinal Caco-2 cells. The highest concentrations of honey (10%) or sugar mixture (5%) tested for quantitative analysis were used to prepare NP dispersions, together with 5% glucose or 5% fructose solution. NP dispersion in MEM was used as a positive control for all cell experiments, because MEM contains fetal bovine serum (FBS), which can interact with ZnO NPs and improve particle dispersion [13]. Figure 8A shows that NP dispersions in honey and in all saccharide solutions significantly inhibited cell proliferation as compared with NPs in DW. The half maximal inhibitory concentration (IC_50_) values were 61.0 ± 2.3, 54.7 ± 1.4, 58.6 ± 2.0, 55.6 ± 1.0, 57.7 ± 2.3 and 112.2 ± 4.8 µg/mL for 5% glucose, 5% fructose, 10% honey and 5% sugar mixture, MEM and DW, respectively. 

On the other hand, when reactive oxygen species (ROS) generation by NPs in different saccharide solutions was evaluated, ROS induction was not significantly influenced by NP interactions with saccharides, except at 50 µg/mL of ZnO NPs (Figure 8B). All saccharide solutions without ZnO NPs did not affect cell proliferation nor ROS generation (Figure S6).

### 2.5. Interaction Effects on Cellular Uptake 

**Figure 9:**
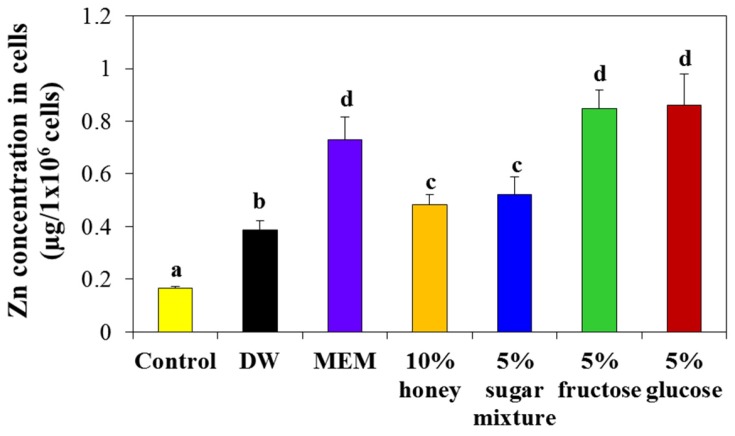
Cellular uptake of ZnO NPs in DW, MEM, or different saccharide solutions in Caco-2 cells after incubation for 6 h, as determined by inductively coupled plasma-atomic emission spectroscopy (ICP-AES). Different lower-case letters (a–d) indicate significant differences among DW, MEM and different saccharide solutions (*p* < 0.05).

The cellular internalized amounts of ZnO NPs in the presence of honey or saccharides were evaluated by measuring total Zn levels inside cells by inductively coupled plasma-atomic emission spectroscopy (ICP-AES). Figure 9 shows that the cellular uptake of NPs was significantly enhanced when NPs were dispersed in 5% fructose or 5% glucose versus that in DW. NP dispersion in MEM also improved cellular uptake but 10% honey and 5% sugar mixture solutions increased cellular uptake only slightly compared with DW control.

### 2.6. Interaction Effects on Intestinal Transport

**Figure 10:**
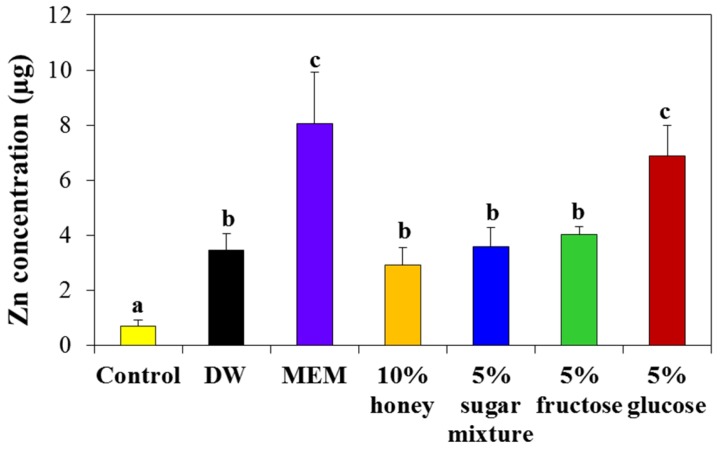
Intestinal transport of ZnO NPs using an in vitro model of human follicle-associated epithelium (FAE). Different lower-case letters (a–c) indicate significant differences among DW, MEM and different saccharide solutions (*p* < 0.05).

The intestinal transport mechanism of ZnO NPs was investigated using an in vitro model of human intestinal follicle-associated epithelium (FAE), representing microfold (M) cells in Peyer’s patches, because our previous research demonstrated that ZnO NPs are primarily transported by M cells [22]. As shown in Figure 10, the transport number of NPs by M cells significantly increased when NPs were dispersed in 5% glucose, while other saccharides did not affect transportation efficiency as compared with NPs in DW. NP dispersed in MEM also promoted the intestinal transport by M cells, in a manner similar to that in 5% glucose.

### 2.7. Interaction Effects on Biokinetics

**Figure 11:**
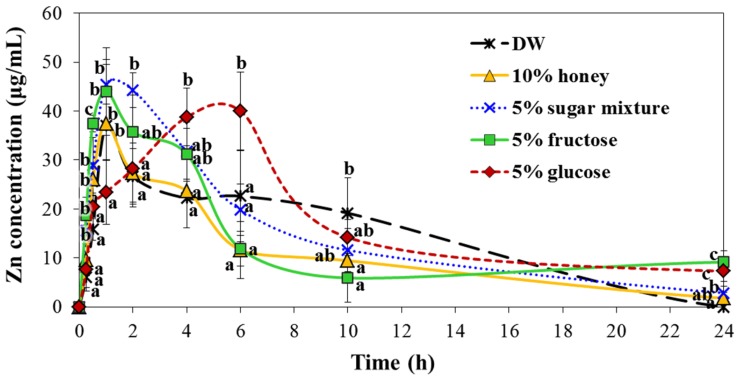
Plasma concentration-time profiles of ZnO NPs suspended in DW or different saccharide solutions after single oral administration to rats. Different lower-case letters (a–c) indicate significant differences between DW and saccharide solutions (*p* < 0.05).

The plasma concentration-time profiles were evaluated after oral administration of ZnO NPs prepared in honey or different saccharide solutions to rats. The result showed that the plasma concentration-time profiles of NPs were highly affected by saccharide type and that the absorption rate in 5% glucose was slower than that in DW (Figure 11). Biokinetic parameters revealed higher area under the plasma concentration-time curve (AUC), half-life (T_1/2_) and mean residence time (MRT) values for 5% fructose and 5% glucose versus DW (Table 1). Based on AUC values, the highest oral absorption (13.4 ± 0.5%) was obtained for 5% glucose, followed by 5% fructose. On the other hand, oral absorption did not significantly increase when NPs were dispersed in 10% honey or 5% sugar mixture versus DW.

## 3. Discussion

The potential interactions between ZnO NPs and saccharides were investigated using 10% acacia honey containing ~4.24% fructose, ~2.96% glucose, ~0.01% sucrose, ~0.01% maltose and other trace nutrients, such as amino acids and minerals. In addition, sugar mixtures composed of equivalent amounts (1%, 2% and 5%) of fructose, glucose, sucrose and maltose without trace nutrients were used for comparative study to determine the role of trace nutrients in NP interactions. Only 5% fructose or 5% glucose solutions, the main two saccharides in acacia honey, were also included to investigate the interactive effects between different saccharides on the interactions.

DLS data revealed that ZnO NPs formed agglomerates or aggregates in aqueous solution (Figure 2 and Figure S1A), although the primary particle size was determined to be ~25.4 ± 9.3 nm (Figure 1). The agglomeration or aggregation tendency of ZnO NPs has been demonstrated in many researches [23,24,25]. In the present study, the hydrodynamic radii of ZnO NPs decreased in the presence of acacia honey or sugar mixture solutions as incubation time increased (Figure 2, Figures S2 and S3), indicating that honey and sugar mixtures contribute to NP dispersion and that ZnO NPs agglomerated, not aggregated in aqueous solution. In particular, sugar mixtures without other minor nutrients were more effective for particle dispersion than acacia honey, suggesting that minor nutrients in the honey differently affect NP interactions. It is worth noting that dramatic decrease in size was observed when NPs were dispersed in 5% fructose or 5% glucose solutions for 1 h (Figure 4 and Figure S4). This may explain a wide application of saccharides as NP dispersants [26,27,28,29]. Similar decrease in hydrodynamic radii of NPs was also observed in MEM (Figure 4 and Figure S4), which was probably due to the role of FBS or albumin in MEM as a dispersant [30,31,32,33]. Notably, the hydrodynamic size of ZnO NPs has been reported to decrease remarkably in the presence of glucose or albumin [13,34,35]. It is known that the size can affect interactions between NPs and food components/biological matrices [22,36,37]. In the present study, the agglomeration tendency of ZnO NPs was reduced by NP-saccharide interactions but NPs with smaller hydrodynamic radii can interact differently with food components.

The zeta potential values of NPs were significantly reduced in both honey and sugar mixture solutions and negative surface charges were induced by 5–10% honey solutions (Figure 3). This result suggests that honey and sugar mixtures interact differently with NPs, probably due to trace nutrients in acacia honey. It is clear that the hydrodynamic radii and surface characteristics of ZnO NPs were affected by saccharides, although the particulate fate of ZnO NPs was not influenced by honey or sugar mixture solutions, as evidenced by their low dissolution property (<1% in all cases).

Quantitative HPLC results demonstrated that the interactions increased as honey or sugar concentrations increased (Figure 6 and Figure 7). However, temperature and incubation time had minimal effects on the interactions. It is worth noting that glucose in acacia honey interacted most actively with ZnO NPs (Figure 6A), although fructose is the most abundant saccharide in honey. This preference was not found in sugar mixture, as no significant differences were observed between saccharide types (Figure 7). This result suggests that minor nutrients in acacia honey, such as amino acids and minerals, play roles in the interactions. Meanwhile, when NPs were incubated with 5% fructose or 5% glucose solutions, significantly high interaction was observed for 5% fructose (~1.3%) than for 5% glucose (~0.8%), which was contrary to that observed for acacia honey or sugar mixtures (Figure 6A and Figure 7A). This implies that the interactive effects between saccharides can affect the interactions, together with the role of trace nutrients. However, NP interactions with saccharides are probably not strong, as no saccharides were detected on the surface of NPs after washing with DW.

When the interactions among ZnO NPs, saccharides and biofluids were evaluated by measuring the hydrodynamic radii of NPs before biological experiments, which reflect the formation of NP-saccharide-biomatrix coronas, the particle size of NPs suspended in 5% fructose or 5% glucose further decreased in MEM and simulated gastric fluid (Figure 4 and Figure S4). This result suggests complex interactions among NPs, food components and biomatrices. In particular, NP suspension in 5% glucose followed by further incubation in simulated gastric fluid was found to contribute most to NP dispersion. The smaller hydrodynamic radii of NPs in gastric fluid as compared with MEM can be explained by their partial dissolution property in gastric fluid (~25%) [35]. It is well known that cell culture medium containing FBS or albumin improve NP dispersion stability [38,39,40,41], which explains the size decrease in MEM, resulted from the interactions. On the other hand, the cellular uptake of ZnO NPs was effectively enhanced by 5% fructose and 5% glucose as compared with DW and similar uptake was found for MEM (Figure 9), which is in-line with the determined hydrodynamic radii of ZnO NPs in MEM (Figure 4 and Figure S4).

The cytotoxicity of ZnO NPs was evaluated after dispersing NPs in the highest concentrations of honey (10%) and sugar mixture (5%) tested and in monosaccharide solutions (5% fructose or 5% glucose) and compared to that in DW or MEM. As shown in Figure 8A, higher inhibition of cell proliferation was induced by ZnO NPs in 5% fructose, 5% glucose, 5% sugar mixture and 10% honey solutions, without statistical differences between saccharide types, than NP dispersion in DW as a control. Furthermore, the cytotoxicity of NPs in MEM was similar to that observed in saccharide solutions. Hence, well dispersed NPs in various saccharide solutions or cell culture medium containing FBS, as evidenced by decreased hydrodynamic radii (Figure 4 and Figure S4), can cause relatively high cytotoxicity in comparison with NPs in DW. However, NP interactions with saccharides were not found to influence ROS generation (Figure 8B). It would appear that ROS was induced by ZnO NPs and not by NP interaction with other components.

When the biokinetic behaviors of ZnO NPs were evaluated after single oral administration to rats, the plasma concentration-time profiles were markedly affected by saccharide type (Figure 11). NPs entered the blood stream more slowly when they were dispersed in 5% glucose than in DW or other saccharide solutions (Figure 11 and Table 1). The highest oral absorption efficiency of NPs encountered was for 5% glucose, which showed ~13.4% absorption (Table 1). Another monosaccharide, 5% fructose also enhanced oral absorption (~9.6%) as compared with DW (7.6%). Interestingly, the amount of ZnO NPs transported by M cells significantly increased when they were dispersed in 5% glucose, as compared with honey or other saccharide solutions (Figure 10), with is in good agreement with our biokinetic results (Figure 11 and Table 1). The absorption efficiency of ZnO NPs in 5% glucose may be related to the role of glucose as a dispersant [21,42], as evidence by DLS data in biological fluids (Figure 4 and Figure S4). Moreover, the intestinal transport efficiency of NPs in 5% glucose, as determined using M cells, would also contribute to their enhanced absorption efficacy following oral administration. These results clearly suggest that the interactions between NPs and saccharides could affect the biokinetic behaviors and oral absorption of ZnO NPs.

## 4. Materials and Methods

### 4.1. Materials and Characterization

ZnO NPs were purchased from Sumitomo (Tokyo, Japan) and dispersed in DW (5 mg/mL) for 30 min prior to experiments. Particle size and morphology were determined by SEM (FEIQUANTA 250 FEG, Hillsboro, OR, USA). XRD patterns for NPs were measured using an X-ray diffractometer (D2phaser, Bruker AXS Inc., Madison, WI, USA) with Ni-filtered CuKα radiation. Zeta potentials and hydrodynamic radii of NPs in aqueous suspension were measured with Zetasizer Nano System (Malvern Instruments, Worcestershire, UK). Materials used were as follow: acacia honey (Dongsuh Food Co., Ltd., Seoul, Republic of Korea), d-(+)-glucose (Sigma-Aldrich, St. Louis, MO, USA), d-(−)-fructose (Sigma-Aldrich), sucrose (Sigma-Aldrich), d-(+)-maltose monohydrate (Sigma-Aldrich). 

### 4.2. Physicochemical Properties of ZnO NPs in Food Matrices or Biomatrices

ZnO NPs were dispersed in 5 mg/mL in acacia honey (1%, 2%, 5% and 10%), sugar mixtures (1%, 2% and 5%), 5% fructose, or 5% glucose solutions. The simulated gastric fluid was prepared by dissolving 2 g sodium chloride (Samchun Chemical Co., Ltd., Republic of Korea) and 3.2 g pepsin (Sigma-Aldrich) in DW and the pH was adjusted to 1.5 with 1 N hydrochloric acid (Duksan Pure Chemicals Co., Ltd., Ansan, Gyeonggi-do, Republic of Korea) and then made up to 1000 mL. Zeta potentials and hydrodynamic radii of NPs in aqueous suspension, food matrices and biofluids were measured with Zetasizer Nano System (Malvern Instruments). Stock solution of ZnO NPs (50 mg/mL in DW) was prepared and stirred for 30 min and diluted to designated concentrations just prior to experiments.

### 4.3. Solubility

For in vitro solubility test, ZnO NPs (5 mg/mL) were dispersed in 10% acacia honey and 5% sugar mixture solution, respectively. After different incubation times at 25 °C, supernatants were collected by ultracentrifugation (16,000× *g*) for 15 min, passed through a syringe filter (pore size 0.45 μm; Advantech, Taipei, Taiwan) and pre-digested with ultrapure nitric acid (HNO_3_) and hydrogen peroxide (H_2_O_2_) as described in “ICP-AES analysis.”

### 4.4. Quantitative Analysis on ZnO NP Interaction with Saccharides 

Acacia honey solutions (1%, 2%, 5% and 10%) were prepared in DW and incubated with 5 mg/mL NPs with shaking at 4, 25 and 40 °C. Sugar mixtures, containing equivalent amounts of fructose, glucose, sucrose and maltose (1%, 2% and 5%) and two monosaccharide solutions (5% fructose and 5% glucose) were also used to evaluate the effect of trace nutrients on the interactions. After designated incubation times (1, 24, 48 h and 7 d), the samples were centrifuged at 23,000× *g* for 1 h. The supernatants were analyzed after washing with distilled and deionized water (DDW) or without washing and then, filtered through syringe filter (Agela Technologies, Wilmington, DE, USA). Saccharide concentrations were quantified by HPLC using a Shimadzu HPLC system (Kyoto, Japan), equipped with RID-10A refractive index detector, on a Hypersil APS-2 column (250 mm × 4.6 mm i.d., 5 µm, 120 Å, Thermo Fisher Scientific, MA, USA). The mobile phase was acetonitrile:water (80:20, *v*/*v*) and flow rate was set at 1 mL/min. Column temperature was maintained at a constant 40 °C and injection volume of sample was 20 µL. Each experiment was repeated three times on separate days.

### 4.5. Cytotoxicity

Human intestinal epithelial Caco-2 cells were purchased from the Korean Cell Line Bank (Seoul, Republic of Korea) and cultured in MEM (Welgene, Gyeongsan, Republic of Korea), under a humidified atmosphere (5% CO_2_/95% air) at 37 °C. The medium was supplemented with 10% heat inactivated FBS, 100 units/mL of penicillin and 100 µg/mL of streptomycin (Welgene). Cells (1 × 10^4^ cells/100 µL) were incubated overnight at 37 °C and then, treated with ZnO NPs dispersed in different saccharide solutions, DW, or MEM for 24 h. In all cell experiments, different saccharide solutions, DW, or MEM without NPs were used as controls. Then, 10 µL of WST-1 solution (Roche, Mannheim, Germany) was added to each well and cells were further incubated for 4 h. Absorbance was measured using a plate reader at 440 nm (SpectraMax^®^ M3, Molecular Devices, Sunnyvale, CA, USA). Cells incubated without NPs were used as controls.

ROS levels were monitored using a peroxide-sensitive fluorescent probe, 2′,7′-dichlorofluorescein diacetate (H_2_DCFDA) (Molecular Probes Inc., Eugene, OR, USA), according to the manufacturer’s guidelines. Cells (1 × 10^4^ cells/100 µL) were incubated with NPs dispersed in different saccharide solutions, DW, or MEM for 24 h under standard condition as described above, washed with phosphate buffered saline (PBS) and incubated with 5 µM H_2_DCFDA for 60 min at 37 °C. After washing with PBS, DCF fluorescence was immediately measured using a fluorescence microplate reader (SpectraMax^®^ M3, Molecular Devices). Excitation and emission wavelengths were 495 and 518 nm, respectively. Cells not treated with NPs were used as controls.

### 4.6. Cellular Uptake

Cells (1 × 10^6^ cells/2 mL) were incubated overnight at 37 °C and treated with ZnO NPs dispersed in different saccharide solutions, DW or MEM. After incubation for 6 h, the cells were washed three times with PBS, treated with 5 mM ethylenediaminetetraacetic acid (EDTA) in PBS for 40 s to detach adsorbed particles on the cell membrane and further washed with PBS. The cells were harvested by scraping and re-suspended in DDW. After centrifugation, the cell pellets were digested and Zn concentrations were determined by ICP-AES (JY2000 Ultrace, HORIBA Jobin Yvon, Longjumeau, France), as described in “ICP-AES analysis” below.

### 4.7. 3D Cell Culture for FAE Model

Human Burkitt’s lymphoma Raji B cells were purchased from the Korean Cell Line Bank and grown in RPMI 1640 medium, supplemented with 10% FBS, 1% non-essential amino acids, 1% l-glutamine, 100 units/mL of penicillin and 100 µg/mL of streptomycin at 37 °C in 5% CO_2_ atmosphere. An in vitro model of human intestinal FAE was prepared as described by des Rieux et al. [43]; Caco-2 cells (1 × 10^6^ cells/well) were grown on upper insert sides in the same manner as described in Caco-2 monoculture system and incubated for 14 days. Raji B cells (1 × 10^6^ cells/well) in DMEM were then added to basolateral insert compartments and these co-cultures were maintained for 5 days. Apical medium of cell monolayers was then replaced with particle suspensions containing ZnO-NPs (62.5 µg/mL) in different saccharide solutions, DW or MEM and incubated for 6 h. The concentrations of transported Zn in basolateral solutions were determined by ICP-AES (JY2000 Ultrace, HORIBA Jobin Yvon).

### 4.8. Animals

Six-week old female Sprague Dawley (SD, 160–170 g) rats were purchased from Koatech. Co (Gyeonggi-do, Republic of Korea). Animals were housed in plastic laboratory animal cages in a ventilated room, maintained at 20 ± 2 °C/60 ± 10% relative humidity with a 12 h light/dark cycle. Water and commercial laboratory complete food for rats were available ad libitum. Animals were environmentally acclimated 7 days before treatment. All animal experiments were performed in compliance with the guideline issued by the Animal and Ethics Review Committee of Seoul Women’s University (IACUC-2016A-3, 29 November 2016), Republic of Korea.

### 4.9. Biokinetic Study

Six female rats per group were administered a single-dose of 300 mg/kg ZnO NPs dispersed in different saccharide solutions or DW via oral gavage and the blood samples were collected via tail vein at several time points (0, 0.25, 0.5, 1, 2, 4, 6, 10 and 24 h) after oral administration. The blood samples were centrifuged at 3000 rpm for 15 min at 4 °C to obtain the plasma. The following biokinetic parameters were estimated using Kinetica software (version 4.4, Thermo Fisher Scientific, Waltham, MA, USA): C_max_, T_max_, AUC, T_1/2_ and MRT.

### 4.10. ICP-AES Analysis

Biological samples were pre-digested with 10 mL of ultrapure nitric acid (HNO_3_) overnight, heated to ~160 °C and 1 mL of hydrogen peroxide (H_2_O_2_) solution was added. The mixtures were heated until the digestion was completed. After dilution with 3 mL of DDW, total Zn concentrations were determined by ICP-AES (JY2000 Ultrace, HORIBA Jobin Yvon, Paris, France).

### 4.11. Statistical Analysis

Results were expressed as means ± standard deviations. A one-way analysis of variance (ANOVA) with Tukey’s Test was performed using SAS Ver.9.4 (SAS Institute Inc., Cary, NC, USA) to determine the significance of differences between experimental groups. Statistical significance was accepted for *p* values < 0.05.

## 5. Conclusions

The interactions between ZnO NPs and saccharides were investigated using acacia honey, sugar mixtures and monosaccharide solutions by measuring the hydrodynamic radii, zeta potentials and solubility and by performing quantitative analysis and evaluating biological responses. The hydrodynamic radii and zeta potential of NPs were found to be highly affected by saccharide type, although their solubility was not affected. Quantitative analysis revealed that NP interactions depended on saccharide type and that minor nutrients in acacia honey might play roles in these interactions. Moreover, interactive effects between saccharides on the interactions were found. On the other hand, cell proliferation was markedly inhibited by NPs in all saccharide types as compared with NPs in DW and cellular uptake of NPs was enhanced by 5% fructose or 5% glucose solutions. The intestinal transport of NPs by M cells and oral absorption were significantly enhanced by 5% glucose, which appears to be associated with their dispersion stability in cell culture medium or gastric fluid, probably attributed to the formation of ZnO NP-saccharide-biomatrix coronas. It can be, therefore, concluded that the complex interactions among food additive NPs, saccharides and biomatrices should be considered to better understand and predict the potential toxicity of NPs in food.

## Figures and Tables

**Table 1:** Biokinetic parameters and oral absorption of ZnO NPs suspended in different saccharide solutions after single oral administration to rats.

Biokinetic Parameters	DW	10% Honey	5% Sugar Mixture	5% Fructose	5% Glucose
C_max_ (mg/L)	37.7 ± 2.2 ^a^	37.5 ± 6.7 ^a^	46.6 ± 3.4 ^b^	44.0 ± 6.9 ^a,b^	40.2 ± 1.2 ^a,b^
T_max_ (h)	1.8 ± 2.0 ^a^	1.0 ± 0.0 ^a^	1.3 ± 0.5 ^a^	1.0 ± 0.0 ^a^	5.3 ± 1.0 ^b^
AUC (h × mg/L)	328.8 ± 27.1 ^a,b^	276.6 ± 18.4 ^a^	364.1 ± 26.5 ^b^	430.4 ± 38.9 ^c^	581.1 ± 20.8 ^d^
T_1/2_	6.3 ± 0.5 ^a^	6.0 ± 0.5 ^a^	5.9 ± 0.9 ^a^	7.4 ± 0.4 ^b^	7.8 ± 0.3 ^b^
MRT (h)	8.5 ± 1.1 ^a^	8.0 ± 1.0 ^a^	8.3 ± 0.6 ^a^	15.7 ± 0.7 ^b^	16.6 ± 0.4 ^b^
Absorption (%)	7.6 ± 0.6 ^a,b^	6.4 ± 0.4 ^a^	8.2 ± 0.5 ^b^	9.6 ± 0.8 ^c^	13.4 ± 0.5 ^d^

Different lower-case letters (a–d) in the same row indicate significant differences between DW and saccharide solutions (*p* < 0.05). C_max_, maximum concentration; T_max_, time to maximum concentration; AUC, area under the plasma concentration-time curve; T_1/2_, half-life; MRT, mean residence time.

## Supplementary Materials

Supplementary materials can be found at http://www.mdpi.com/1422-0067/19/2/486/s1.
