# Supplementary material for: Effects of Interactions between ZnO Nanoparticles and Saccharides on Biological Responses
Source: Int J Mol Sci. 2018 Feb 6;19(2):486. doi: 10.3390/ijms19020486 (PMC5855708; doi:10.3390/ijms19020486)
Supplement: Supplementary file 1 [file ijms-19-00486-s001.pdf]

Effects of Interactions between ZnO Nanoparticles and Saccharides on Biological Responses

Mi-Ran Go, Jin Yu, Song-Hwa Bae, Hyeon-Jin Kim and Soo-Jin Choi *

Major of Food Science & Technology, Department of Applied Food System, Seoul Women's University, Seoul 01797, Korea; miran8190@naver.com (M.-R.G.); ky5031@swu.ac.kr (J.Y.); songhwa29@naver.com (S.-H.B.); kimhj043@naver.com (H.-J.K.)

* Correspondence: sjchoi@swu.ac.kr; Tel.: +82-2-970-5634; Fax: +82-2-970-5977

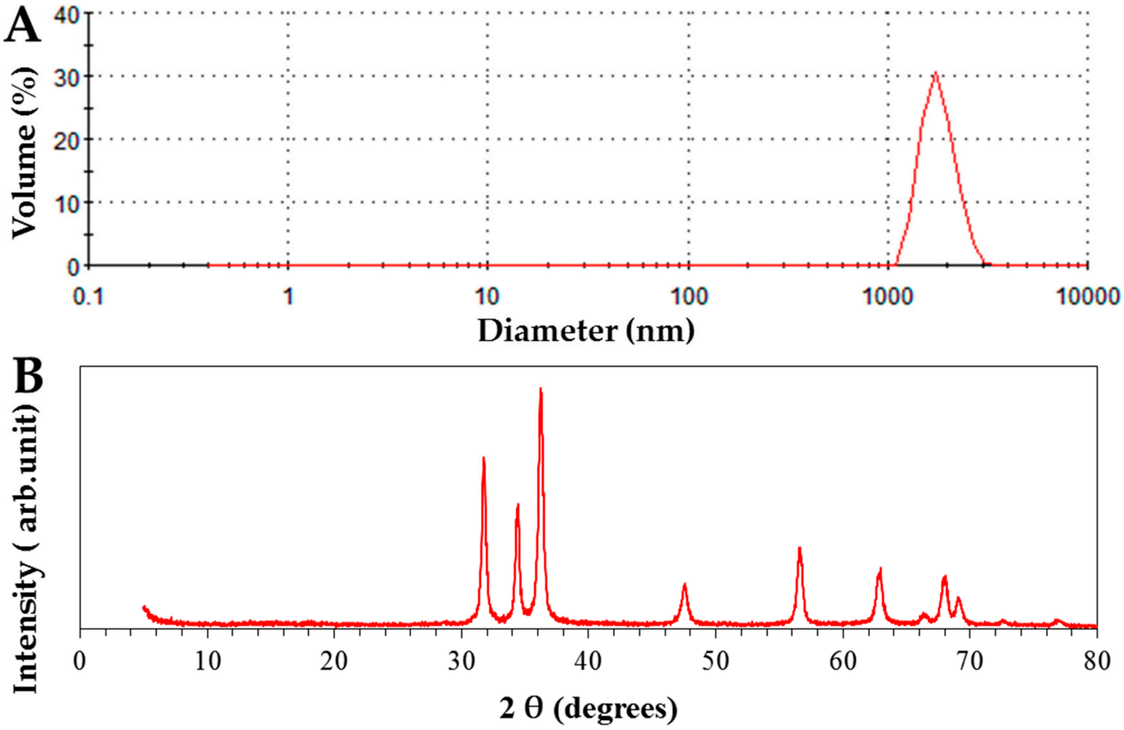

Figure S1. (**A**) Hydrodynamic particle size distribution and (**B**) powder X-ray diffraction (XRD) pattern of ZnO NPs.

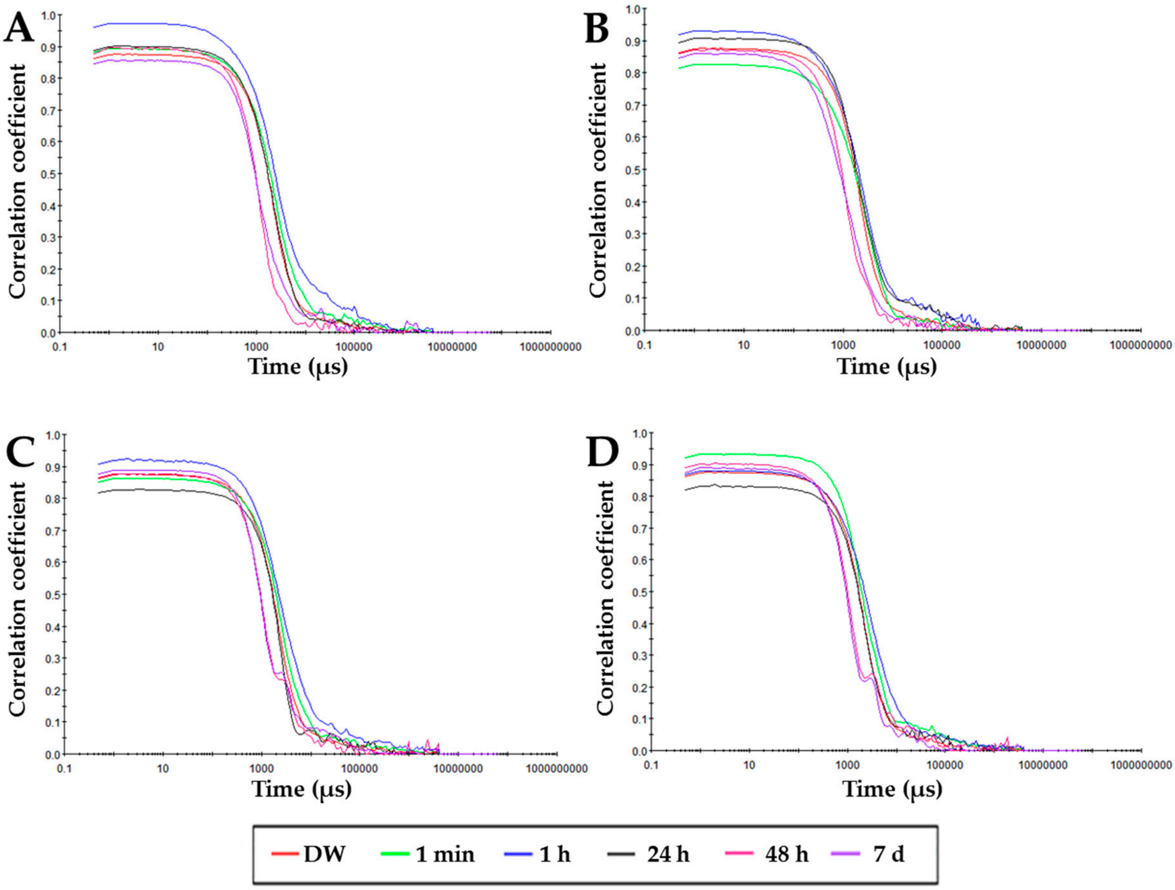

Figure S2. Correlation functions of DLS studies of ZnO NPs in (**A**) 1%, (**B**) 2%, and (**C**) 5%, and (**D**) 10% honey.

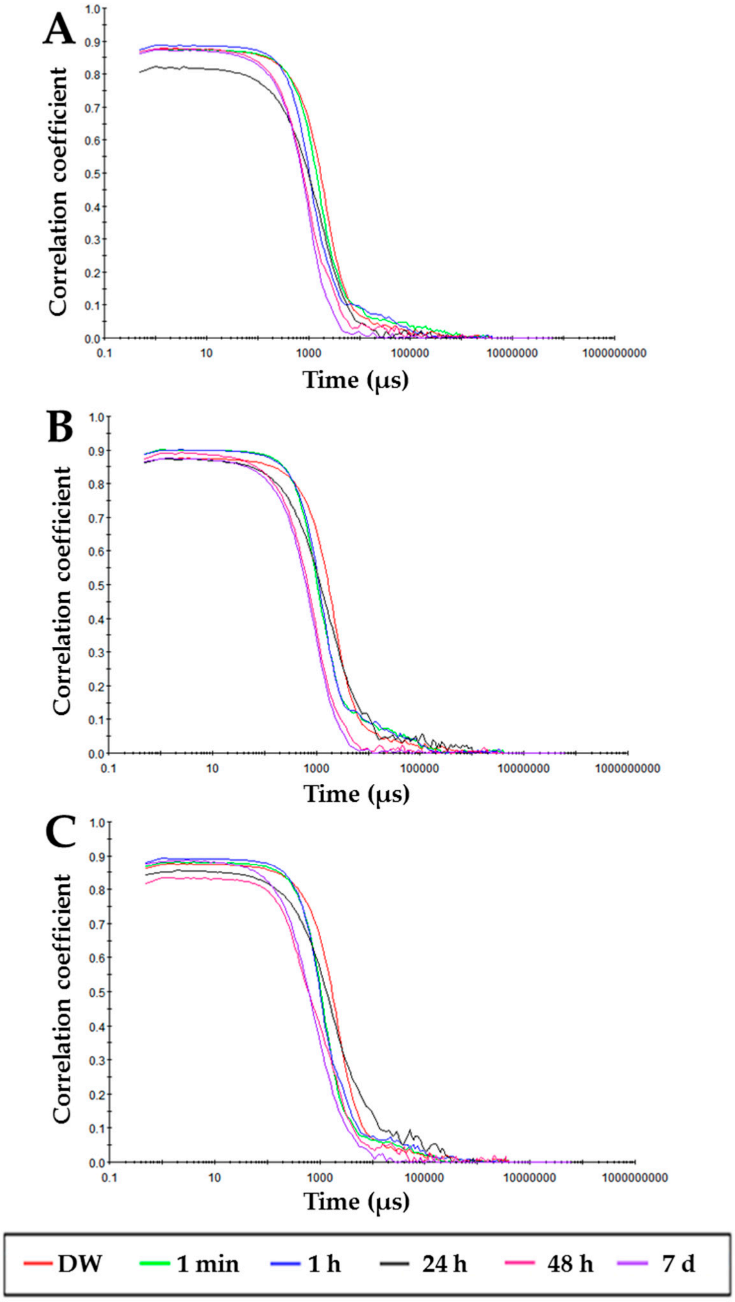

Figure S3. Correlation functions of DLS studies of ZnO NPs in (**A**) 1%, (**B**) 2%, and (**C**) 5% sugar mixture.

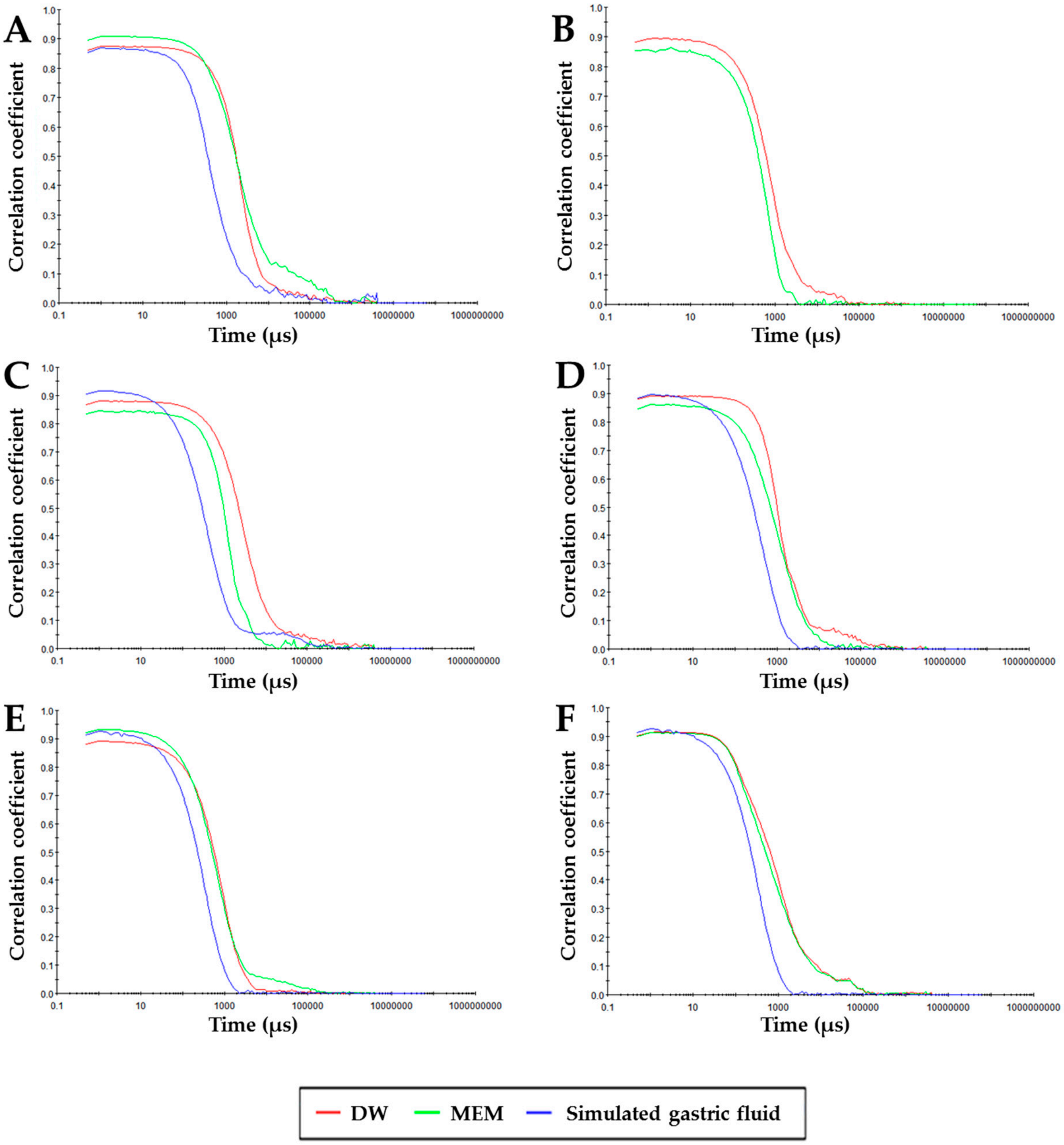

Figure S4. Correlation functions of DLS studies of ZnO NPs dispersed in (**A**) DW, (**B**) minimum essential medium (MEM), (**C**) 10% honey, (**D**) 5% sugar mixture, (**E**) 5% fructose, and (**F**) 5% glucose for 1 h, and then, measured in biological fluids (MEM and simulated gastric fluid) or DW as a control.

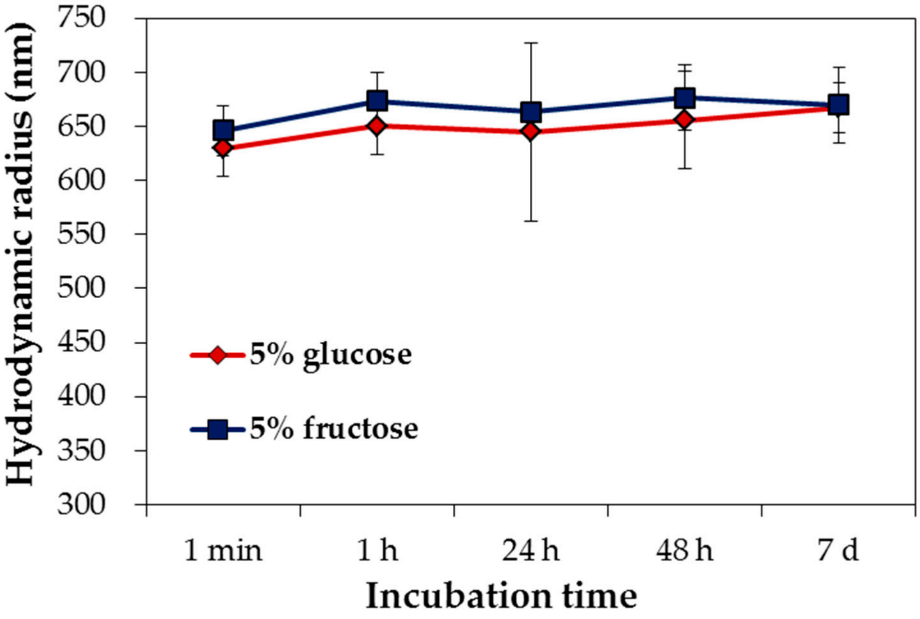

Figure S5. Hydrodynamic radii of ZnO NPs in 5% glucose and 5% fructose solutions for 7 days.

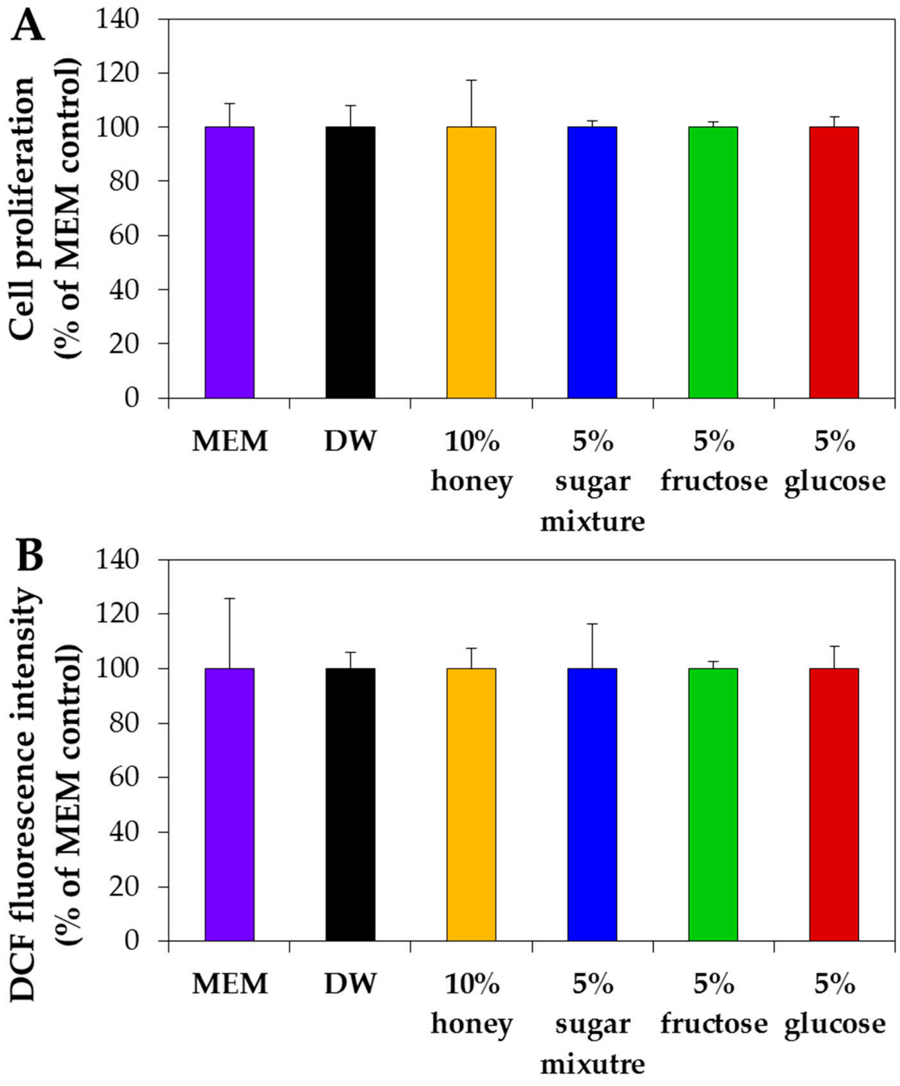

Figure S6. Effect of DW or different saccharide solutions on (**A**) Caco-2 cell proliferation and (**B**) intracellular ROS generation after 24 h.
